# Chromophore attachment to fusion protein of streptavidin and recombinant allophycocyanin α subunit

**DOI:** 10.1080/21655979.2017.1321282

**Published:** 2017-05-19

**Authors:** Jing Wu, Huaxin Chen, Peng Jiang

**Affiliations:** a Key Laboratory of Experimental Marine Biology, Institute of Oceanology, Chinese Academy of Sciences, Qingdao, China; b Laboratory for Marine Biology and Biotechnology, Qingdao National Laboratory for Marine Science and Technology, Qingdao, China; c University of Chinese Academy of Sciences, Beijing, China

**Keywords:** Allophycocyanin, Chromophore attachment, Chromophorylation rate, Immunofluorescence assay, Phycobiliprotein

## Abstract

The fusion protein (SLA) of streptavidin and allophycocyanin α subunit (holo-ApcA) was biosynthesized in *Escherichia coli* by a dual plasmid system. The recombinant SLA, purified by affinity chromatography, showed spectral properties similar to natural allophycocyanin α subunit (ApcA). Spectral and Zinc staining analysis indicated that the recombinant SLA covalently bound phycocyanobilin (PCB). To improve chromophorylation rate of recombinant SLA, an *in vitro* chromophore attachment reaction system was established, which contained partially chromophylated SLA, PCB and lyase CpcS. Spectral analysis showed that PCB bound to the recombinant SLA rapidly during the reaction. The chromophorylation rate of SLA was improved from 21.1% to 86.5%. Immunofluorescence assay showed that SLA with high chromophorylation rate had higher detection signal. Thus, *in vitro* chromophore attachment is an effective way to improve the chromophorylation rate of recombinant phycobiliprotein.

## Introduction

Phycobiliproteins are light-harvesting antennae found in cyanobacteria, red algae and cryptomonas.^1^ Allophycocyanin (APC), C-phycocyanin (CPC), and phycoerythrins (PE) are the 3 most common phycobiliproteins in cyanobacteria. Each phycobiliprotein contains α and β subunits.^2^ The phycobiliprotein covalently binds linear tetrapyrrole phycobilins and absorbs visible light ranging from 500 nm to 700 nm. Being water-soluble fluorescent proteins, phycobiliproteins have widespread applications as fluorescent labels.

In recent years, the biosynthesis pathway for cyanobacteria phycobiliproteins has been elucidated. By co-expression of genes for the apoprotein, lyases and genes for phycocyanobilin biosynthesis in *Escherichia coli* cells, the pathway of phycobiliproteins can be re-constructed in *E. coli* cells. Several type of phycobiliproteins, including holo-CpcA, holo-ApcA and holo-ApcB, had been biosynthesized in *E. coli*.^3,4^ Thespectroscopic properties of recombinant phycobiliproteins were similar to those of the same protein produced endogenously in cyanobacteria.^5-7^ The recombinant phycobiliproteins could be fluorescent labels serving as a substitute for native phycobiliproteins.

Recombinant phycobiliproteins reconstituted in *E. coli*, however, are only partially chromophorylated. A considerable portion of phycobiliprotein lacks phycobilin. Tooley et al.^7^ constructed the pathway for CpcA from *Synechocystis* sp. PCC6803 in *E. coli*. About a third of the apo-CpcA was converted to holo-CpcA. Biswas et al.^5^ recreated the biosynthetic pathway for several type of phycobiliproteins from *Synechococcus* sp strain PCC 7002 in *E. coli*. The chromophorylation rates were estimated to be from 17.4% to 71.9%. The low chromophorylation rate was not likely to be limited by availability of heme. Rather, it was supposed to be due to low expression level or activity of the lyases, Ho1 and PcyA.^7^

In this paper, heme oxygenase (Ho1) and ferredoxin oxidoreductase (PcyA) responsible for the conversion of cellular heme to PCB, together with SLA and lyase CpcS were co-expressed in *E. coli* by a dual plasmid system. The recombinant cell produced partially chromophorylated SLA. We performed an *in vitro* chromophore attachment reaction using purified partially chromophorylated SLA and PCB in the presence of CpcS, with an aim to improve the chromophorylation rate. Spectral properties and the performance in immunofluorescence assay of the recombinant SLA were further analyzed.

## Results

### *In vivo* and *in vitro* chromophore attachment

**Figure 1.**
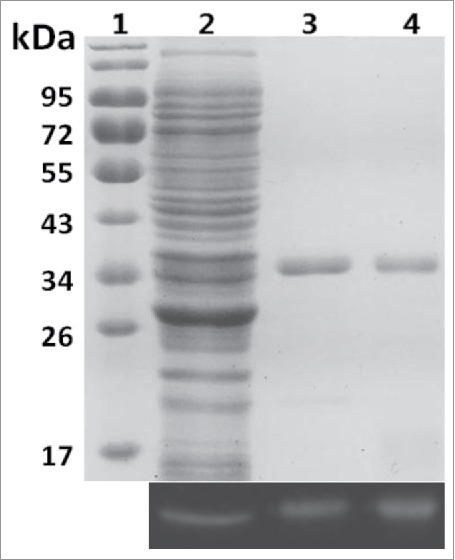
SDS-PAGE and Zn^2+^-UV fluorography of SLA: Lane 1, marker; Lane 2, whole protein in bacteria extract; Lane 3, purified SLA biosynthesized *in vivo*; Lane 4, purified SLA further chromophorylated *in vitro*.

**Figure 2.**
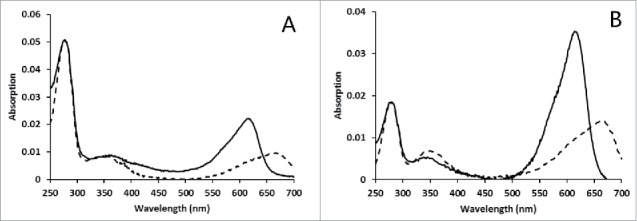
Absorption spectra of SLA biosynthesized *in vivo* and further chromophorylated *in vitro*. Proteins were treated with 8 M urea (pH 1.5) for 30 min. Absorption spectra of SLA biosynthesized *in vivo* (A) and SLA further chromophorylated *in vitro* (B) with (dashed lines) or without (solid lines) urea treatment were then recorded.

To produce the fusion protein SLA, dual plasmid expression systems were used. The plasmid pCDFDuet-SLA-CpcS for overexpress the SLA and lyase CpcS, together with the plasmid pRSFDuet-Ho1-PcyA for overexpress Ho1 and PcyA, were co-transformed into *E. coli*. The cells turned blue after 18 h induction by IPTG. The *E. coli* cells were harvested, disrupted and the His-tagged fusion protein SLA was purified with a metal affinity column. The protein was analyzed via SDS-PAGE (Fig. 1). The major Coomassie blue-stained band of 34 kDa corresponded to the calculated molecular mass of SLA. Zn^2+^-induced fluorescence on denaturing gels confirmed the covalent attachment of chromophore. SLA had an absorption maximum at 614 nm and an emission maximum at 633 nm (Table 2). The absorption and emission spectra are similar to those of native ApcA.^8,9^ After denaturation in acidic urea solution (8 M, pH 1.5), the denatured SLA gave maximal absorption at 660 nm (Fig. 2), indicating the correct attachment of PCB to the apoprotein. The characteristic absorption at 614 nm of SLA was significantly lower than the absorption at 280 nm, with an A_614_/_280_ ratio of 0.5 (Fig. 2, Table 2). The chromophorylation rate was calculated to be 21.1%, indicating that the recombinant purified protein was partially chromophorylated.

**Figure 3.**
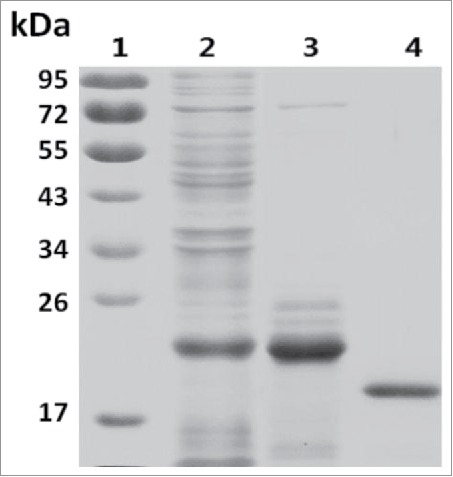
SDS-PAGE of lyase: Lane 1, marker; Lane 2, whole protein in bacteria extract; Lane 3, purified CpcST; Lane 4, purified CpcS.

To promote attachment of PCB to SLA, we performed an *in vitro* chromophore attachment reaction using purified SLA, CpcS and PCB. To prepare CpcS, CpcST was expressed in *E. coli* and purified with a metal affinity column. Then the protein was digested with TEV protease and His-tag was removed. SDS-PAGE showed that a single band for CpcS corresponded to 20 kDa, suggesting the His-tag had been removed (Fig. 3).

**Figure 4.**
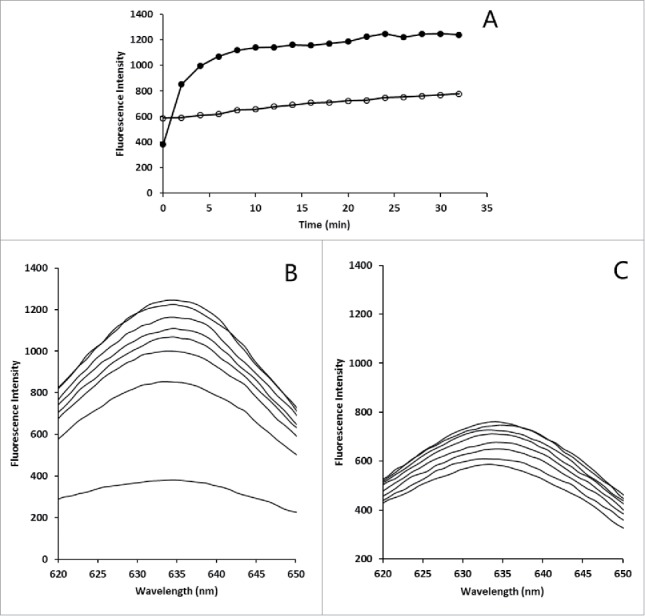
*In vitro* PCB attachment to partially chromophorylated SLA. (A) Increase in emission maximum over time at the λmax of the final product. Closed circles: chromophore attachment reaction system with CpcS; open circles: chromophore attachment reaction system without CpcS. (B) Fluorescence spectra of the *in vitro* chromophore attachment assay with CpcS. (C) Fluorescence spectra of the *in vitro* chromophore attachment assay without CpcS.

The fluorescence spectra during the chromophore attachment reaction were recorded at 2 min intervals (Fig. 4). Purified SLA exhibited an emission maximum at 633 nm. During the attachment reaction, the fluorescence intensity of the mixture continued to increase over time, indicating that PCB attached to SLA during the time course (Fig. 4). In the presence of CpcS, the fluorescence intensity of protein increased rapidly and the attachment reaction was nearly complete in 10 min. In the absence of CpcS, however, slight increase of the fluorescence intensity occurred. The result indicates that SLA has ability of autocatalytic chromophore attachment.

**Figure 5.**
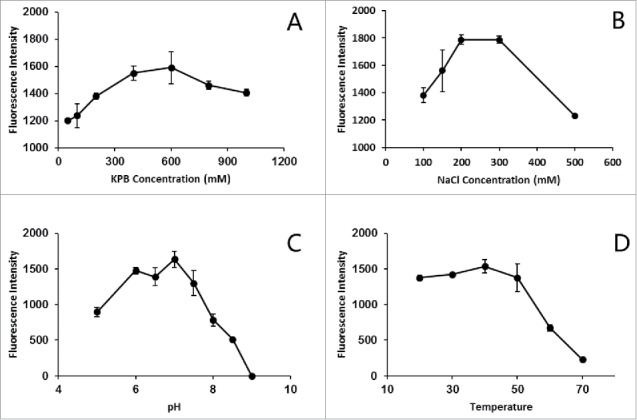
Optimization of attachment conditions containing ionic strength, pH and temperature. Each data point is the average of 3 replicates with error bar representing SD.

Several investigations were then made to determine the optimal reaction conditions (Fig. 5). The lyase tends to precipitate in low ionic strength buffers, and its activity is affected by pH, temperature and other factors, such as metal ions.^10^ Finally, the optimization conditions (600 mM KPB, 250 mM NaCl, pH 7.0, temperature 37°C and incubation time of 30 min) were determined.

After the reaction product was purified, equal amounts of SLA were loaded on an SDS-PAGE gel and Zn^2+^-enhanced fluorescence was examined (Fig. 1). The SLA further chromophorylated *in vitro* had stronger fluorescence than the *in vivo* chromophorylated SLA. Spectral analysis showed that the ratio of A_614_/_280_ of SLA was elevated near to 2 (Fig. 1). In addition, the chromophorylation rate was improved from 21.1% to 86.5% (Table 2). These results demonstrated that SLA was efficiently chromophorylated during the chromophore attachment reaction.

### Immunofluorescence assay using SLA as fluorescent label

**Figure 6.**
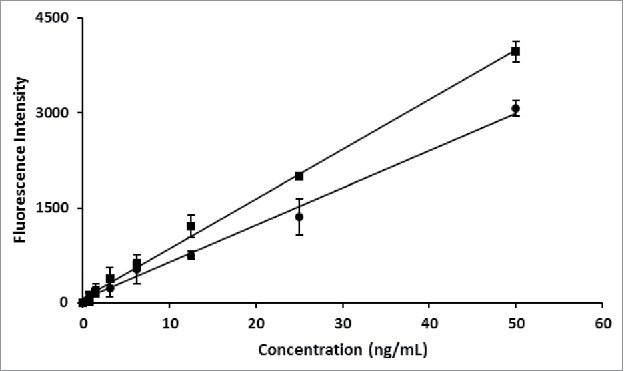
Determination of the AFP concentration by using SLA in sandwich immunoassay. Squares: SLA biosynthesized in *E. coli*; Diamonds: SLA further chromophorylated *in vitro*. Each data point is the average of 3 replicates with error bar representing SD.

Streptavidin has an extremely high affinity for biotin (K_d_ = 10^−14^–10^−16^) and there is no significant effect on biotin-binding ability when streptavidin is cross-linked to proteins or other detecting reagents.^11^ SLA as fluorescent label in immunofluorescence assay was analyzed. α fetal protein (AFP), which is a serological marker of liver cancer, was used as a model analyte. The result showed that the standard curve for AFP had a good linearity in the range of 0.02 ng/mL to 50 ng/mL (Fig. 6). Meanwhile, the slope of the standard curve for SLA further chromophorylated *in vitro* was higher than that for SLA chromophorylated biosynthesized *in vivo*, indicating that chromophorylation rate of SLA is critical to its performance in immunofluorescence assay.

## Discussion

In this work, fusion protein of streptavidin and allophycocyanin α subunit was successfully biosynthesized, which has spectral properties similar to natural ApcA.^12^ Based on its intense fluorescence and biotin-binding ability, SLA could be a promising fluorescent label in immunofluorescence assay. However, the recombinant SLA was partially chromophorylated. The unchromophorylated SLA could not be separated from the chromophorylated SLA by conventional chromatography method, and thus would lead to low fluorescence signal in immunofluorescence assay. The limited chromophorylation is universal issue for phycobiliproteins biosynthesized in *E. coli*.^5,7,13^ Previous studies showed that chromophorylation rate ranged from 17.4% to 71.9%, depending on the type of phycobiliproteins.^5^ The limited chromophorylation of recombinant proteins may be due in part to codon usage unfavorable for *E. coli* to generate large amounts of Ho1, PcyA and lyases.^7^ In our previous work, we found that addtion of δ-aminolevulinic acid and hemin to culture medium did not efficiently increase the chromophorylation rate. In addition, HPLC analysis showed that there was no detectable PCB in recombinant *E. coli* cells (data not shown). These results suggested that limited chromophorylation was related to PCB deficiency in *E. coli* and cannot be resolved by expression conditions optimizations.

With an attempt to improve chromophorylation rate of recombinant SLA, we established an *in vitro* attachment reaction system, which contained partially chromophylated SLA, PCB and lyase CpcS. Autocatalytic attachment of PCB to apo-SLA occurred (Fig. 4), which is similar to the reports that autocatalytic binding of PCB to ApcA may occur.^9,14^ This antocatalytic reaction was slow and incomplete and lyase was necessary for efficient attachment of PCB to SLA. Through *in vitro* attachment reaction, the chromophorylation rate of recombinant SLA was significantly improved (Table 2). The immunofluorescence assay showed that this high chromophorylation SLA exhibited higher fluorescence signal, compared with the low chromophorylation SLA biosynthesized *in vivo*.

To summarize, chromophore attachment *in vitro* is an effective way to improve the chromophorylation rate of recombinant SLA, which will contribute to the application of recombinant phycobiliproteins in immunofluorescence assay.

## Materials and methods

### Biosynthesis of SLA

The *apcA* and *cpcS* genes from *Thermosynechococcus elongatus* BP-1 were amplified by PCR. The primers for PCR amplification were showed in Table 1. The *sa* gene coding for core streptavidin was artificially synthesized in Shanghai Sunnybio Biotechnology Co. Ltd., and was fused to *apcA* by using recombinant PCR. A 78 bp nucleotides linker (Table 1) was used to link the 2 genes. This fusion gene, denoted as SLA, was digested with the BamHI and SacI and ligated into similarly digested pCDFDuet-1 vector, yielding plasmid pCDFDuet-SLA. The *cpcS* gene was digested with NdeI and XhoI and ligated into similarly digested pCDFDuet-SLA, yielding plasmid pCDFDuet-SLA-CpcS. The *Ho1* gene coding for heme oxygenase was digested with the BamHI and SacI and ligated into similarly digested pRSFDuet-1 vector, yielding plasmid pRSFDuet-Ho1. The *PcyA* gene coding for ferredoxin oxidoreductase was digested with the NdeI and XhoI and ligated into similarly digested pRSFDuet- Ho1 vector, yielding plasmid pRSFDuet-Ho1-PcyA. The final constructed plasmids were sequenced to check for the validity of gene sequences.

**Table 1.** List of primers for PCR amplification.

Genes	Primers	Restriction enzymes	
*apcA*	5′AAGGATCCGATGAGTATCGTCACGAA3′	*BamHI*	
	5′GCGAGCTCCTAGCTCATTTTTCCGAT3′	*SacI*	
*cpcS*	5′GTACATATGGATGCAATGGAATT3′	*NdeI*	
	5′AGCGATATCCTACCAGCCACAAAATTG3′	*XhoI*	
*linker*	5′GGATCCGCCGAAGCGGCCGCAAAAGAAGCTGCGGCCAAGGAAGCAGCTGCGAAAGAAGCCGCAGCTAAGGCGGAATTC3′

**Table 2.** Spectroscopic properties of SLA biosynthesized *in vivo* and further chromophorylated *in vitro*.

	Absorption		Fluorescence		Chromophorylation rate	
Sample	λ_max_ (nm) (Qvis/UV)	ε (M^-1^cm^-1^)	λ_max_ (nm)	Φ_F_	*In vivo*	*In vitro*
SLA	614/280	105600(±300)	633	0.33±0.4	21.1%(±10.6%)	86.5 %(±9.6%)

Each data point is the average of three replicates with error bar representing SD.

The pCDFDuet-SLA-CpcS and pRSFDuet-Ho1-PcyA expression vectors were co-transformed into *E. coli* BL21 (DE3). A single colony was cultured in 6 mL of LB medium with 100 μg/mL spectinomycin and 100 μg/mL kanamycin at 37°C overnight. The bacterial culture was transferred into 300 mL of TB medium containing corresponding antibiotics and incubated with shaking at 37°C. When the cell density reached OD_600_ of 0.8, the cultures were induced with 1 mM IPTG at 18°C for 18 h. The cells were subsequently harvested by centrifugation at 6000 × g for 10 min. After washed with distilled water, the cells were suspended in 30 mL of binding buffer (20 mM sodium phosphate and 20 mM imidazole, pH 7.4) and lysed by ultrasonication. The recombinant His-tagged protein was isolated from the supernatant Ni^2+^-chelating affinity column. Then the protein solution was further purified using a Sephadex G25 column with desalting buffer (10 mM sodium phosphate and 150 mM NaCl, pH 7.4).

### Preparation of CpcS and PCB

To construct the expression vector of CpcS, standard procedures were used. The primers used to amplify the *cpcS* gene from *Thermosynechococcus elongatus* BP-1 were 5′GCGGAATTCGGAAAACCTGTATTTTCAGGGTGTGTGCATAGGTATGGAC3′ and 5′GCGAAGCTTCAGGAGTTGGCGGGTTGCGTCA3′, which were designed to contain a TEV protease recognition site (7 amino acid sequence of Glu-Asn-Leu-Tyr-Phe-Gln-Gly). The amplified DNA fragment denoted as CpcST was digested with EcoRI and HindIII and then ligated into similarly digested pRSFDuet-1 to yield pRSFDuet-CpcST. The vector consisted of the 6 His-tag sequence at the N-terminus of coding region for CpcST. The final constructed plasmid was sequenced to check for the validity of gene sequences.

The expression vector described above was transformed into *E. coli* BL21 and the transformants were selected by 100 μg/mL kanamycin. The cells were cultured in TB medium supplemented with antibiotics. After induction with 1 mM IPTG for 18 h at 18°C, the cells were harvested, disrupted, and purified by Ni^2+^-chelating affinity column. Then the high-concentration imidazole was removed using a Sephadex G25 column.

To remove His-tag on CpcST, TEV protease digestion was performed with purified CpcST (1 mg) and His-tagged TEV protease (0.02 mg) in TEV buffer (50 mM NaH_2_PO_4_, 150 mM NaCl, 1 mM EDTA, 1 mM DTT, pH 8.0) for 6 h at 16°C. Then CpcS was purified by Ni^2+^-chelating affinity column, and CpcS flew out directly during sample injection. The protein concentration was determined with the commercial protein assay kit. The proteins were analyzed by SDS-PAGE.

The plasmid pRSFDuet-Ho1-PcyA was used for PCB biosynthesis. Singly transformed *E. coli* BL21 cells containing pRSFDuet-Ho1-PcyA was selected by 100 μg/mL kanamycin. Then the cells were cultured and induced with 1 mM IPTG for 18 h at 18°C. The *E. coli* cells were harvested and resuspended with 100% methanol. PCB was released after extracting for 2 h at 4°C. After centrifugation at 8000 × g for 30 min at 4°C, the supernatant was transferred into a rotary evaporator, and PCB was concentrated by spin vacuum and stored at −20°C. PCB concentrations were determined spectroscopically using an excitation coefficient of 35.5 mM^−1^ cm^−1^ at 660 nm.^15^

### *In vitro *chromophore attachment

Chromophore attachment *in vitro* was performed to improve chromophorylation rate of purified SLA. For attachment assay, purified SLA (10 μM), purified CpcS (5 μM) and PCB (20 μM) were mixed in 1 mL of 500 mM potassium phosphate (pH 7.0) containing 150 mM NaCl. The CpcS free reaction system was used as a control. After rapid mixing had been performed, the fluorescence spectra during the attachment reaction was measured at 2 min intervals using the fluorescence spectrophotometer. Emission spectra were measured from 620 to 650 nm with excitation at 590 nm.

To optimize conditions for *in vitro* chromophore attachment reaction, several investigations were made as follows. PCB was mixed with SLA and CpcS in KPB (50–1000 mM, pH 5.0–9.0) containing NaCl (100–500 mM). The fluorescence of the mixtures was measured, after the mixture was incubated at 20–70°C for 1 h in the dark.

Incubated under the optimized conditions, the mixture was then concentrated by ultrafiltration and washed 3 times with binding buffer (20 mM sodium phosphate and 20 mM imidazole, pH 7.4). SLA was purified by Ni^2+^-affinity chromatography.

### Analysis of SLA

Protein concentrations were determined with a Bradford protein assay kit (Tiangen). The proteins were analyzed by 12% sodium dodecyl sulfate-polyacrylamide gel electrophoresis (SDS-PAGE). Before Coomassie staining, the resolved gels were soaked in 20 mM zinc acetate for 10 min at room temperature. The Zn^2+^-enhanced fluorescence was observed by UV fluorography.

The absorption spectra were analyzed by a UV-1801 spectrophotometer (Rayleigh) using a 0.5 cm path length cuvette. The spectra were recorded from 250 nm to 700 nm at a bandwidth of 1 cm with a scan speed of 240 nm/min. The fluorescence emission spectra were analyzed by an F-4500 fluorescence spectrophotometer (Hitachi). The excitation wavelength was 590 nm and the emission spectra were recorded from 620 nm to 650 nm. The emission and excitation slit widths were set to 5 nm with a scan speed of 240 nm/min.

The proteins were further denatured in 8.0 M urea (pH 1.5). The absorption spectra were as described above. To calculate the chromophorylation rate, the concentration of PCB was divided by the concentration of purified SLA.

### Sandwich immunofluorescence assay using SLA

The wells of 96-well black plate (Thermo) were coated overnight at 4°C with 100 μL of 5 μg/mL anti-AFP capture antibody (Fitzgerald), diluted in 50 mM sodium bicarbonate buffer (pH 9.6). The wells were washed 3 times with 200 μL of PBST (0.01M PBS containing 0.05% Tween-20, pH 7.4). Then 100 μL of 5% skim milk powder was added into the wells for 2 h at 37°C. After washing as above, a series of AFP concentration were added to the capture antibody coated wells. The immunoreaction was allowed to proceed for 1 h with continuous shaking at 37°C followed by washing. Then 100 μL of 4 μg/mL biotinylated detection antibody (Fitzgerald) was added into each well and further incubated at 37°C for 1 h with continuous shaking. Afterwards, unbound biotinylated anti-AFP antibody was removed by washing the wells with 200 μL of PBST. 100 μL of SLA was added into cells and further incubated at 37°C for 1 h. The wells were then washed 3 times with 200 μL of PBST. Finally, the fluorescence intensities at 640 nm with excitation at 590 nm was measured by a M100 Pro microplate reader (Tecan Trading AG).

## References

[1] Wilbanks SM, Glazer AN Rod structure of a phycoerythrin II-containing phycobilisome. 1. Organization and sequence of the gene cluster encoding the major phycobiliprotein rod components in the genome of marine *Synechococcus* SP. WH8020. J Biol Chem 1993; 268:1226-35; PMID:8419325 8419325

[2] MacColl R Cyanobacterial phycobilisomes. J Struct Biol 1998; 124:311-34; PMID:10049814; https://doi.org/10.1006/jsbi.1998.4062 10049814

[3] Yang Y, Ge BS, Guan XY, Zhang WJ, Qin S Combinational biosynthesis of a fluorescent cyanobacterial holo-alpha-allophycocyanin in *Escherichia coli*. Biotechnol Lett 2008; 30:1001-4; PMID:18224279; https://doi.org/10.1007/s10529-008-9644-2 18224279

[4] Liu S, Chen Y, Lu Y, Chen H, Li F, Qin S Biosynthesis of fluorescent cyanobacterial allophycocyanin trimer in *Escherichia coli*. Photosynth Res 2010; 105:135-42; PMID:20607408; https://doi.org/10.1007/s11120-010-9574-4 20607408

[5] Biswas A, Vasquez YM, Dragomani TM, Kronfel ML, Williams SR, Alvey RM, Bryant DA, Schluchter WM Biosynthesis of Cyanobacterial Phycobiliproteins in *Escherichia coli*: Chromophorylation Efficiency and Specificity of All Bilin Lyases from *Synechococcus* sp. Strain PCC 7002. Appl Environ Microb 2010; 76:2729-39; https://doi.org/10.1128/AEM.03100-09 PMC2863458 20228104

[6] Chen H, Lin H, Li F, Jiang P, Qin S Biosynthesis of a stable allophycocyanin beta subunit in metabolically engineered *Escherichia coli*. J Biosci Bioeng 2013; 115:485-9; PMID:23266116; https://doi.org/10.1016/j.jbiosc.2012.11.008 23266116

[7] Tooley AJ, Cai YPA, Glazer AN Biosynthesis of a fluorescent cyanobacterial C-phycocyanin holo-alpha subunit in a heterologous host. P Natl Acad Sci USA 2001; 98:10560-5; https://doi.org/10.1073/pnas.181340998 PMC58505 11553806

[8] Cohenbazire G, Beguin S, Rimon S, Glazer AN, Brown DM Physico-Chemical and Immunological Properties of Allophycocyanins. Arch Microbiol 1977; 111:225-38; PMID:65163; https://doi.org/10.1007/BF00549359 65163

[9] Hu IC, Lee TR, Lin HF, Chiueh CC, Lyu PC Biosynthesis of fluorescent allophycocyanin alpha-subunits by autocatalytic bilin attachment. Biochemistry 2006; 45:7092-9; PMID:16752899; https://doi.org/10.1021/bi052067a 16752899

[10] Zhao KH, Su P, Li J, Tu JM, Zhou M, Bubenzer C, Scheer H Chromophore attachment to phycobiliprotein beta-subunits: phycocyanobilin:cysteine-beta84 phycobiliprotein lyase activity of CpeS-like protein from *Anabaena* Sp. PCC7120. J Biol Chem 2006; 281:8573-81; PMID:16452471; https://doi.org/10.1074/jbc.M513796200 16452471

[11] Inouye S, J-i Sato, Sasaki S, Sahara Y Streptavidin-Aequorin Fusion Protein for Bioluminescent Immunoassay. Biosci Biotech Bioch 2011; 75:568-71; https://doi.org/10.1271/bbb.100798 21389603

[12] MacColl R, Eisele LE, Menikh A Allophycocyanin: Trimers, monomers, subunits, and homodimers. Biopolymers 2003; 72:352-65; PMID:12949826; https://doi.org/10.1002/bip.10437 12949826

[13] Yi JJ, Xu D, Zang XN, Yuan DY, Zhao BR, Tang L, an YN, Zhang XC Lyase activities of heterologous CpcS and CpcT for phycocyanin holo-beta-subunit from Arthrospira platensis in *Escherichia coli*. J Ocean U China 2014; 13:497-502; https://doi.org/10.1007/s11802-014-2161-0

[14] Scheer H, Zhao KH Biliprotein maturation: the chromophore attachment. Mol Microbiol 2008; 68:263-76; PMID:18284595; https://doi.org/10.1111/j.1365-2958.2008.06160.x 18284595 PMC2327270

[15] Glazer AN, Fang S Chromophore content of blue-green algal phycobiliproteins. J Biol Chem 1973; 248:659-62; PMID:4630849 4630849

